# Effects of various wet environments on the characteristics of the dust cake deposited on the surface of filter media

**DOI:** 10.1038/s41598-023-44429-4

**Published:** 2023-10-10

**Authors:** Shihang Li, Yihan Lin, Muze Han, Hao Liu, Jiang Shao, Xiaoyu Tan, Yuchen Luo, Rongting Huang

**Affiliations:** 1 Jiangsu Key Laboratory of Coal-Based Greenhouse Gas Control and Utilization, Carbon Neutrality Institute, China University of Mining and Technology, Xuzhou, 221116 China; 2 School of Environment Science and Spatial Informatics, China University of Mining and Technology, Xuzhou, 221116 China; 3 Jiangsu Engineering Research Center of Dust Control and Occupational Protection, School of Safety Engineering, China University of Mining and Technology, Xuzhou, 221116 China

**Keywords:** Environmental sciences, Energy science and technology, Engineering

## Abstract

The effect of wet environments on the dust cake of filter media was studied. The collapse angles of dust particles and the collapse angles between dust particles and filter media increase with increasing dust moisture content, relative humidity, and spray rate. The smallest growth rate of collapse was observed under dust moisture content, while the largest growth rate occurred under the spray rate condition. The collapse angles between dust particles and filter media of coated filter media were smaller compared to those of mechanical filter media under different wet environments. The dust cake drag coefficients of both filter media initially increase and then decrease with an increase in the dust moisture content, decrease with the acceleration of the relative humidity, and show a pattern of first decreasing and then increasing as the spray rate increases. The dust loading capacity of both filter media follows an opposite trend to that of the dust cake drag coefficients.

## Introduction

Rock tunneling and coal mining often generate significant amounts of fine dust, posing a serious threat to occupational health and safety^1,2^. Traditional dust removal methods have their advantages and disadvantages. Ventilation-based dust removal, in general, leads to environmental pollution at the back of the roadway and necessitates supplementary dust removal techniques. Spraying, coal seam water injection, and wet dust collectors consume large quantities of water and are less effective in capturing respiratory dust. Foam dust suppression materials require continuous investment. Therefore, traditional dust removal methods alone cannot adequately address the issue of dust pollution in coal mine tunnels and rock tunnel construction, particularly when it comes to effectively purifying respiratory dust.

By utilizing a cartridge dust collector, no water is consumed, there is no secondary pollution, and the dust collection efficiency can reach up to 98% when combined with a wall-mounted duct. This helps to address the limitations of traditional dust collection methods^3–5^. Cartridge dust collectors are increasingly being adopted in roadways and tunnels. During the excavation process of coal mine workings, cartridge dust collectors often encounter challenges such as increased filtration resistance, poor pulse-jet cleaning, and reduces air volume, which negatively impact their performance in the wet environment of coal mines^3,6^. Underground coal mine sites are exposed to various wet environments. The first type of wet environment is caused by dust moisture content: coal bodies may contain moisture due to the presence of groundwater. Additionally, water injection methods are often used to pre-wet the coal body in most coal mines, resulting in a certain amount of moisture in the dust^7^. The second type of wet environment is determined by relative humidity. The relative humidity in underground coal mines can be over 80%^8^. The third type of wet environment is associated with spray rate, as water spraying is commonly implemented in underground coal mining to reduce dust levels and lower equipment temperatures^9^. Cartridge dust collectors mainly use filter media to remove dust and purify the air. The performance of the filter media is influenced by various factors. Previous studies have focused on optimizing the filter media performance through adjustments to fiber diameter, filling density, fold structure, and other parameters^10–12^. Some researchers have investigated the effects of wet environments, including relative humidity, dust moisture content, and spray rate, on filter media performance. However, no comparative study has been conducted to assess the impact of the three wet environments, such as dust moisture content, relative humidity and spray rate.

Gupta et al. studied the impact of relative humidity, particle hygroscopicity, and particle size on the dust loading capacity of mechanical filter media using NaCl and Al_2_O_3_ particles^13^. The results demonstrated that relative humidity played a significant role in increasing the dust loading capacity of the filter media for both hygroscopic and non-hygroscopic particles when the relative humidity levels were below the deliquescence point. On the basis of Hajra et al.^14^, relative humidity and temperature can influence the loading performance of glass filter media, where higher humidity levels lead to a greater pressure drop. Similar to the conclusion of Gupta et al.^13^, when relative humidity is high, it condenses into liquid droplets, reducing the available pore space of the filter media and resulting in higher pressure drop at deliquescence point. Miguel et al.^15^ investigated the influence of relative humidity on the density of the dust cake formed on the surface of the filter media. The findings indicated that an increase in relative humidity resulted in the formation of more compact particles and a rise in the density of the dust cake. Joubert et al.^16^. indicated that the effect of pleated filter media and relative humidity is dependent on the plugging stage, suggesting that the findings for flat media may not be directly applicable to pleated media. Joubert et al.^17^ then focused on the implication of relative humidity on the filtration characteristics of the dust cake, which forms in successive layers during the plugging phase under specific relative humidity conditions, with the pressure drop $(\Delta P)$ across each layer of particles depended on its equilibrium with the surrounding relative humidity. Thus, it can be inferred that relative humidity conditions inevitably affects filtration and the deposition of the dust cake on the filter media surface. However, the relative humidity conditions investigated in these studies were below saturation humidity, preventing the formation of a liquid film on the surfaces of the particles. The wet environment of underground construction sites is complex and requires further discussion.

Li et al.^18^ researched the effect of dust moisture content on the performance of mechanical filter media, coated filter media and waterproof filter media and measured the filtration pressure drop $(\Delta P)$, dust cake filtration resistance ($\Delta {P}_{\text{C}}$), dust cake porosity, adhesion force, as well as other parameters under different levels of dust moisture content. According to the results, both $\Delta P$ and $\Delta {P}_{\text{C}}$ initially increased and then decreased with increasing dust moisture content. Furthermore, exhibited a decreasing trend followed by an increasing trend, which was notably different from the influence of relative humidity. Li et al.^3^ explored the effect of water mist on the filtration performance of the dust cake, and observed that the efficiency of pulse-jet cleaning decreased as the water mist level increased due to the enhanced capillary force, which led to increased adhesion of dust particles and difficulty in removing them. The study suggest that an optimal amount of water mist can prolong the filtration cycle of the filter media, thereby reducing the frequency of pulse-jet cleaning of the filter media. Water mist can increase friction and adhesion between dust particles, thereby loosening the dust cake structure and reducing the $\Delta {P}_{\text{C}}$. Instead, excessive water mist can result in the formation of more liquid bridges between particles, leading to an increase in $\Delta {P}_{\text{C}}$. Under spray conditions, Nie et al.^19^ performed a numerical simulation to examine the dynamic changes between dust particles and droplets. The results revealed that droplets wrap dust particles better at velocities ranging from 20 to 30 m/s, and the optimal droplet size is 14–30 μm. Different wet environments, such as dust moisture content, relative humidity and spray rate, may exert distinct influences on filtration performance, and the influences of each wet environment on dust cake is inconclusive.

This paper aims to investigate the effects of various wet environments on the characteristics of the dust cake deposited on the surface of filter media. A testing system for analyze the dust cake parameters in various wet environments was established. The impacts of wet environments on the collapse angles between dust particles and filter media and collapse angles between dust particles and filter media were researched. The changes in the dust cake drag coefficients and the dust loading capacity under various wet environments were evaluated. This paper contributes to a deeper understanding of the influence of various wet environments on the dust cake deposited on the surface of filter media.

## Experimental method

### Experimental setup

**Figure 1:**
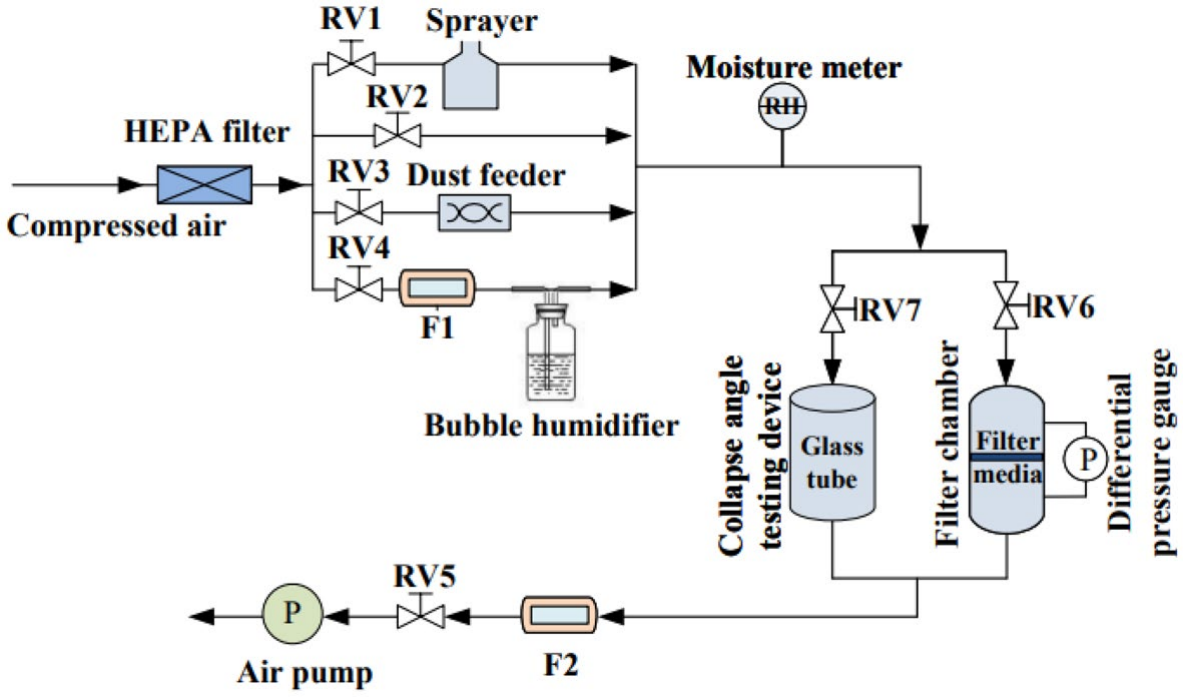
Experimental setup.

A testing system (Fig. 1) was established to measure the influence of dust moisture content, relative humidity, and spray rate on the parameters of dust cake deposited on the surface of filter media. This system includes of regulating valves RV1-RV7 (controlling the opening and closing of the individual lines in the experimental system), flow meters F1 and F2 (measuring pipeline flow, TSI 4040, American), an air pump (offering negative pumping pressure), high efficiency filters, a dust feeder (generating specific concentrations of dust, Topas SAG-410, Germany), a moisture meter (measuring the humidity level of the air, AR 827, China), a differential pressure instrument (testing the filtration resistance between the two sides of the filter media, TSI 5825, American), a bubble humidifier (regulating the wet level of the air), a sprayer (generating fine droplets of air, NE-C900, Japan), a filter chamber (diameter being 5.5 cm, and filter area being 23.8 cm^2^), a collapse angle test device (measuring collapse angle, the diameter and length being 12 cm and 30 cm, respectively), a micrometer balance (weighing, JJ2023BC), etc.

In the experiments, mechanical filter media and coated filter media were selected for testing. The mechanical filter media has a fiber diameter of about 1.5 × 10^–5^ m and a thickness of about 0.5 mm. The coated filter media is made by laminating a polytetrafluoroethylene (PTFE) microporous film on the surface of the coated filter media, which has the pore size of 1 × 10^–5^–6 × 10^–5^ m. The specific parameters and scanning electron microscope images of filter media, as well as parameters of particle have been presented in the authors’ previous study^20^, specific parameters showed in Fig. S1 and Table S1.

### Setting up wet environments

The setting methods of various wet environments can be found in the authors’ previous study^20^, also showed in Table S2.

### Test procedures and methods

#### Test of collapse angles of dust particles

1/3 volume of fly ash was added into the glass tube under various wet environments. (1) For dust moisture content, the dust with specific moisture content was added into the glass tube. Then, both ends of the glass tube were closed. The glass tube was rotated every 5 min for 1 h. (2) For relative humidity, dry dust was added to the glass tube. The air pump was turned on with RV2, RV4, RV5, and RV7 opened, and RV1, RV3, and RV6 closed. The RH was adjusted to 50%, 60%, 70%, 80%, 90% and 100%, respectively. The glass tube was rotated every 5 min for 1 h to ensure that it reached the dynamic balance condition, and then both ends of the glass tube were closed. (3) For spray, dry dust was added to the glass tube. The air pump was turned on with RV1, RV2, RV5, and RV7 opened, and RV3, RV4, and RV6 closed. Spray rate of 2, 4, 6, 8, 10 and 12 mL/s were provided, respectively. The glass tube was rotated every 5 min for 1 h to ensure that it reached the dynamic balance condition, and then both ends of the glass tube were closed.

**Figure 2:**
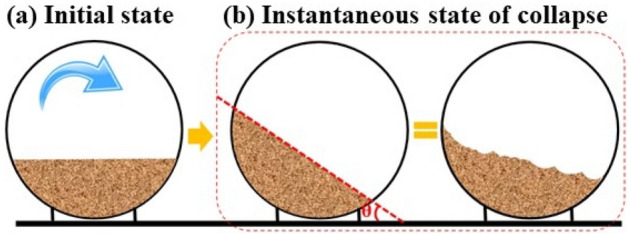
An illustration of the collapse angle test method.

The collapse angle test method is shown in Fig. 2. First, keep the upper plane of the dust inside the glass tube parallel to the horizontal line after processing the dust inside the glass tube according to the above steps. Then, roll the glass tube slowly in the direction depicted in Fig. 2a. The rotation speed of the glass tube was 3 r/min. The dust inside the glass tube gradually inclines, as shown in Fig. 2b. The dust inside the glass tube suddenly collapses when the angle reaches a certain value. At this time, the angle formed by the deviation of the upper surface of the dust from the horizontal line represents the angle at which the dust particles collapse. The angle of the upper plane of the dust deviates from the horizontal line is the collapse angles of dust particles^21^.

#### Test of collapse angles between dust particles and filter media

The test method of the collapse angles between dust particles and filter media is similar to that collapse angles of dust particles. The test filter media should be tightly fastened to the inner wall of the glass tube in advance and then repeat the steps in Test of collapse angles of dust particles.

### Test of dust cake drag coefficients

The testing system presented in Fig. 1 was utilized for the experiment. The test filter media was first weighed and then placed into the filter chamber. The air pump was turned on with RV2, RV5, and RV6 opened, and RV1, RV3, RV4, and RV7 closed. The initial filtration velocity was set to 4.5 cm/s using RV2, RV5, and RV6. The initial $\Delta {P}_{\text{F}}$ of the mechanical filter media and coated filter media were recorded.

Secondly, dust was produced at the speed of 0.1 g/min for 5 min. The $\Delta {P}_{\text{F}}$ and the value of the F2 were recorded in actual time. (1) For dust moisture content, dust samples with different moisture contents were added into the dust feeder. Afterward, the dust feeder and the air pump were opened with RV2, RV3, RV5, and RV6 opened and RV1, RV4, and RV7 closed. (2) For relative humidity dry dust was added into the dust feeder. Then, the dust feeder and the air pump were opened with RV2, RV3, RV4, RV5, and RV6 opened and RV1 and RV7 closed. The relative humidity was measured by the hygrometer in real time. (3) For spray, dry dust was added into the dust feeder. Then, the dust feeder and the air pump were opened with RV1, RV2, RV3, RV5, and RV6 opened and RV4 and RV7 closed.

According to Eq. (1), the total pressure drop recorded by the differential pressure gauge can be divided into filter media pressure drop and dust cake pressure drop^22–24^.

1 $$\Delta P=\Delta {P}_{\text{F}}+\Delta {P}_{\text{C}}=K{v}_{f}+S{v}_{f}$$

where $\Delta P$ is the total pressure drop, Pa, $\Delta {P}_{\text{F}}$ is the filter media pressure drop, Pa, $\Delta {P}_{\text{C}}$ is the dust cake pressure drop, Pa, *K* is the coefficient of resistance of filter media, Pa s/m, *S* is the dust cake drag coefficient, Pa s/m, ${v}_{f}$ is the filtration velocity, m/s.

### Test of dust loading capacity

After the loading process test in Test of dust cake drag coefficients, the test filter media was removed from the filter chamber and put into the oven. The oven temperature was set to 110 °C for 1 h. The dried filter media was placed in a desiccator and cooled for 15 min before being weighed. The weight of drying filter media minus the initial weight is the dust loading capacity.

## Results and discussion

### The effect of wet environments on the collapse angles of dust particles

**Figure 3:**
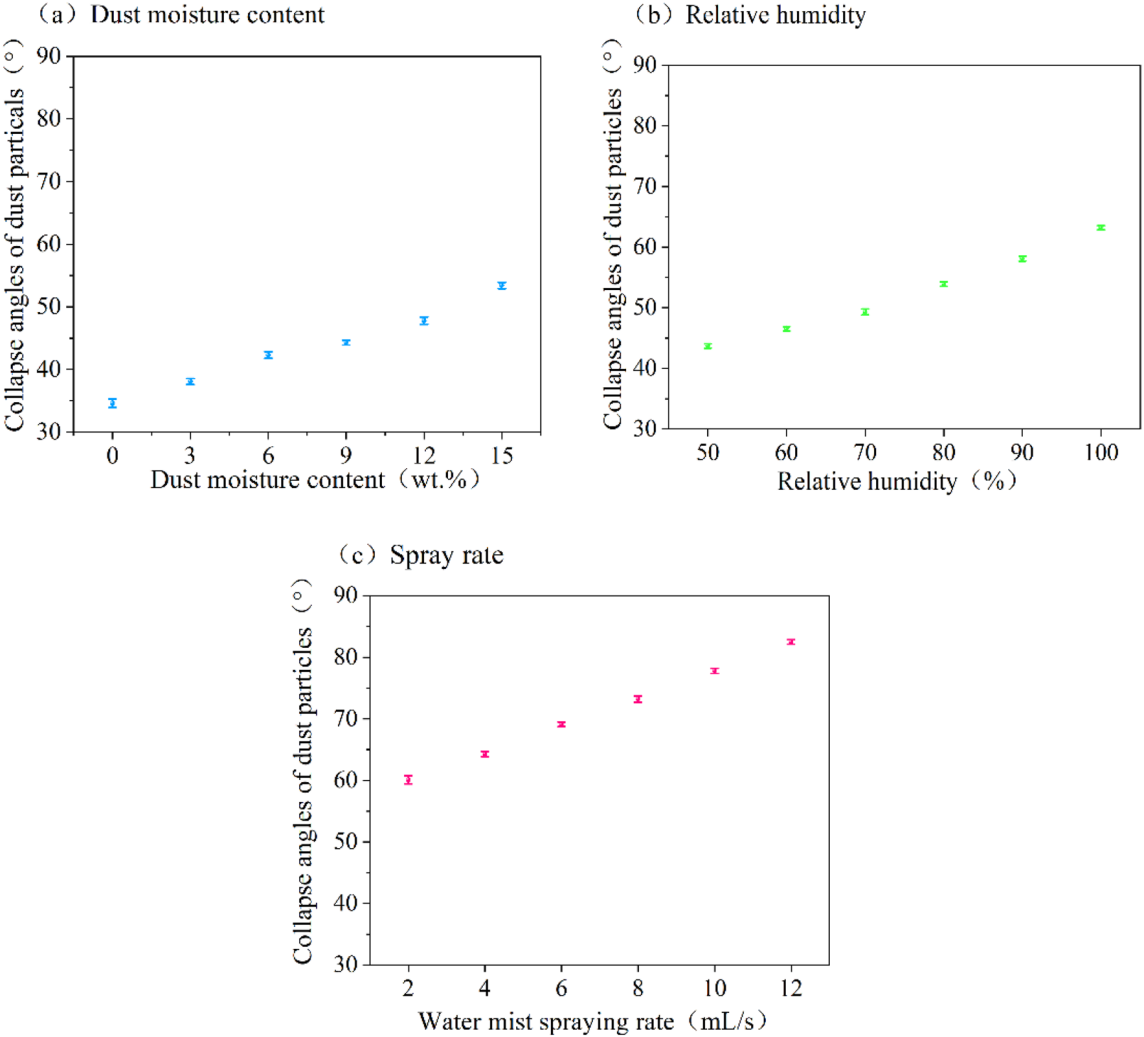
Collapse angles of dust particles under different wet environments.

The collapse angles of dust particles in various wet environments are shown in Fig. 3. The collapse angles of dust particles increase with the dust moisture content rises. When the dust moisture content is 0 wt%, the collapse angles of dust particles is the smallest, measuring 34.6°. When the dust moisture content is 15 wt%, the collapse angles of dust particles is the largest, measuring 53.4°. According to the study by Li et al.^18^, the results can be explained by the fact that when dust contains water, it adheres to the water’s surface, thereby enhancing inter-particle adhesion. As the dust moisture content increases, more particles carry moisture on their surfaces, leading to an increase in inter-particle adhesion, further resulting in an elevated the collapse angles of dust particles.

The collapse angles of dust particles under different relative humidity conditions are shown in Fig. 3b. When the relative humidity is 50%, the collapse angles of dust particles is the smallest, measuring 43.7°. When the relative humidity is 100%, the collapse angles of dust particles is the largest at 63.2°. The increase in collapse angles of dust particles with rising relative humidity can be attributed to an increase of dust particles forming liquid bridges, and enhanced dust adhesion, resulting in an increase in collapse angles of dust particles with the rise in RH. Pei et al.^25^ observed the influence of relative humidity on the dust deposition patterns on the surface of filter media through experiments. The results proved that under the influence of relative humidity, dendrite structures can form between particles, leading to different collapse angles.

The collapse angles of dust particles at different spray rate are presented in Fig. 3c. It is observed that collapse angles of dust particles increased with an increase in spray rate. At spray rate of 2 mL/s, the collapse angles of dust particles is the lowest, measuring 60.1°, while at spray rate of 12 mL/s, it is the largest, measuring 82.5°. Spraying is considered equivalent to relative humidity after saturation, causing dust particles to collapse at wider angles than under relative humidity condition. In the presence of relative humidity, the condensation of water vapor is influenced by the gaps between dust particles. Wider gaps between dust particles facilitate the occurrence of “capillary condensation” as water vapor can easily reach these spaces. As a result, water vapor condensation occurs in tighter particle gaps. During spraying, the behavior of droplets are no longer controlled by particle gaps. Liquid water can be directly attached to the surfaces of dust particles, forming liquid bridges more rapidly and easily. Additionally, as the spray rate increases, more small droplets are formed within per unit of space, leading to an increased formation of liquid bridges between dust particles. Consequently, both the inter-particle liquid bridge force and inter-particle adhesion force intensify, resulting in a larger collapse angles of dust particles.

As shown in Fig. 3, the collapse angles of dust particles observed under dust moisture content conditions are lower than that observed under relative humidity conditions. This result is due to the treatment of dust with water, followed by a 2-month period of sealing to ensure even mixing of sprayed water within the dust particles, but not all of the water adheres to the surface of the dust particles, some may remain in the fine pores of the dust, reducing the probability of liquid bridge formation. The adhesion between particles is also small, resulting in a relatively small collapse angles of dust particles. Wet environments, such as dust moisture, relative humidity and spray rate, can cause the formation of liquid bridges between dust particles. According to the data, it can be observed that under spray conditions, the collapse angle between dust particles is maximum, and the formation of liquid bridges is no longer constrained by the gaps between particles and can occur rapidly on the particle surface. The number of liquid bridges formed is influenced by the different wet environments and their respective values, which, in turn, affect the inter-particle forces and result in varying collapse angles of dust particles.

### The influence of wet environments on the collapse angles between dust particles and filter media

**Figure 4:**
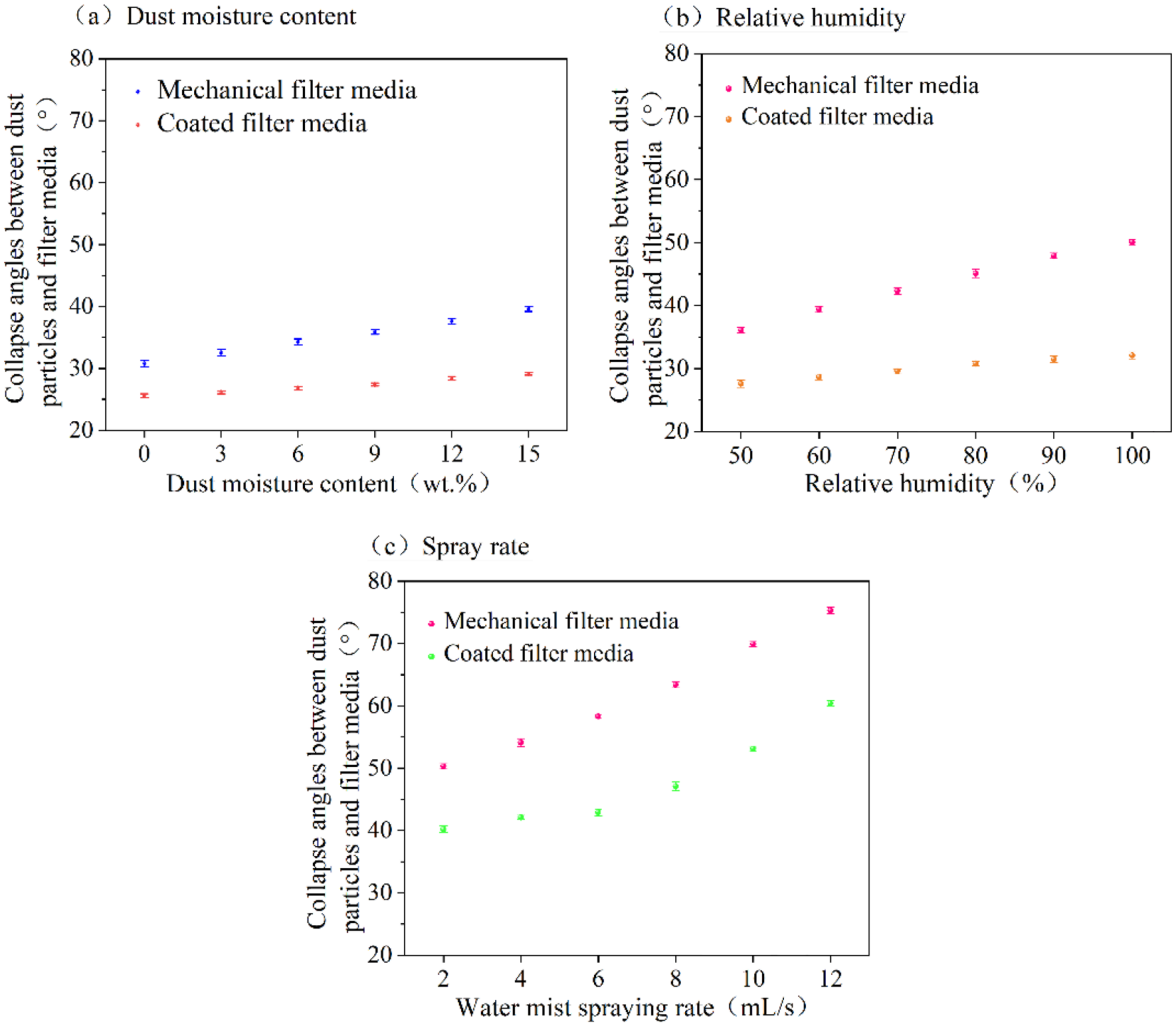
CADF-M and CADF-C under different wet environments.

The adhesion of dust particles to the surface of filter media can vary depending on wet environment, then affects the adhesion state of dust particles and the efficiency of pulse-jet cleaning. The collapse angles between dust particles and mechanical filter media (CADF-M) and the collapse angles between dust particles and coated filter media (CADF-C) under wet environments were shown in Fig. 4. The CADF-M and CADF-C increase slowly as dust moisture content increases. When the dust moisture content is 0 wt%, the CADF-M and CADF-C are the lowest, 30.8° and 25.6°, respectively. When the dust moisture content is 15 wt%, the CADF-M and CADF-C are the largest, 39.6° and 29.1° As shown in Fig. 4b, CADF-M and CADF-C increase as relative humidity increases, whereas CADF-M rises more rapidly. At 50% relative humidity, CADF-M and CADF-C are the smallest, 36.1° and 27.6°, respectively; at 100% relative humidity, CADF-M and CADF-C are the largest, 50.1° and 32.1°, respectively. Regarding the spray rate, Fig. 4c indicates that both CADF-M and CADF-C increase as spray rate increases. At an spray rate of 2 mL/s, CADF-M and CADF-C are the smallest, at 50.3° and 40.2°, respectively; at spray rate of 12 mL/s, CADF-M and CADF-C are the greatest, at 75.3° and 60.4°, respectively. At lower spray rate, the CADF-M and CADF-C are less variability; at higher spray rate, due to the increase number of liquid bridges formed between the dust particles and the filter media, the adhesion between the dust particles and the filter media becomes stronger, leading to a higher rate of change in collapse angles between dust particles and filter media.

As shown in Fig. 4, under different dust moisture content, relative humidity, and spray rate conditions, collapse angles between dust particles and filter media is increasing, while CADF-M is greater than CADF-C. Furthermore, it is observed that changes in the wet environment have a relatively smaller impact on CADF-C compared to CADF-M, primarily because mechanical filter media has a rougher surface than coated filter media. The presence of moisture leading to a higher capillary force on the surface of mechanical filter media, which, in turn, increases dust adhesion between dust particles and the mechanical filter media, ultimately resulting in a larger collapse angle. Under the dust moisture content, relative humidity, and lower spray rate conditions, the amplification of CADF-C is relatively small due to the smoother surface of the coated filter media, which facilitates the removal of dust from its surface. However, if the spray rate is extremely high, such as when water wets the roadway wall or when there is a large amount of spray involved, the CADF-C increases dramatically. Then the force between dust particles and coated filter media increases, making it more difficult to remove dust from the coated filter media surface.

### The effect of wet environments on dust cake drag coefficients

**Figure 5:**
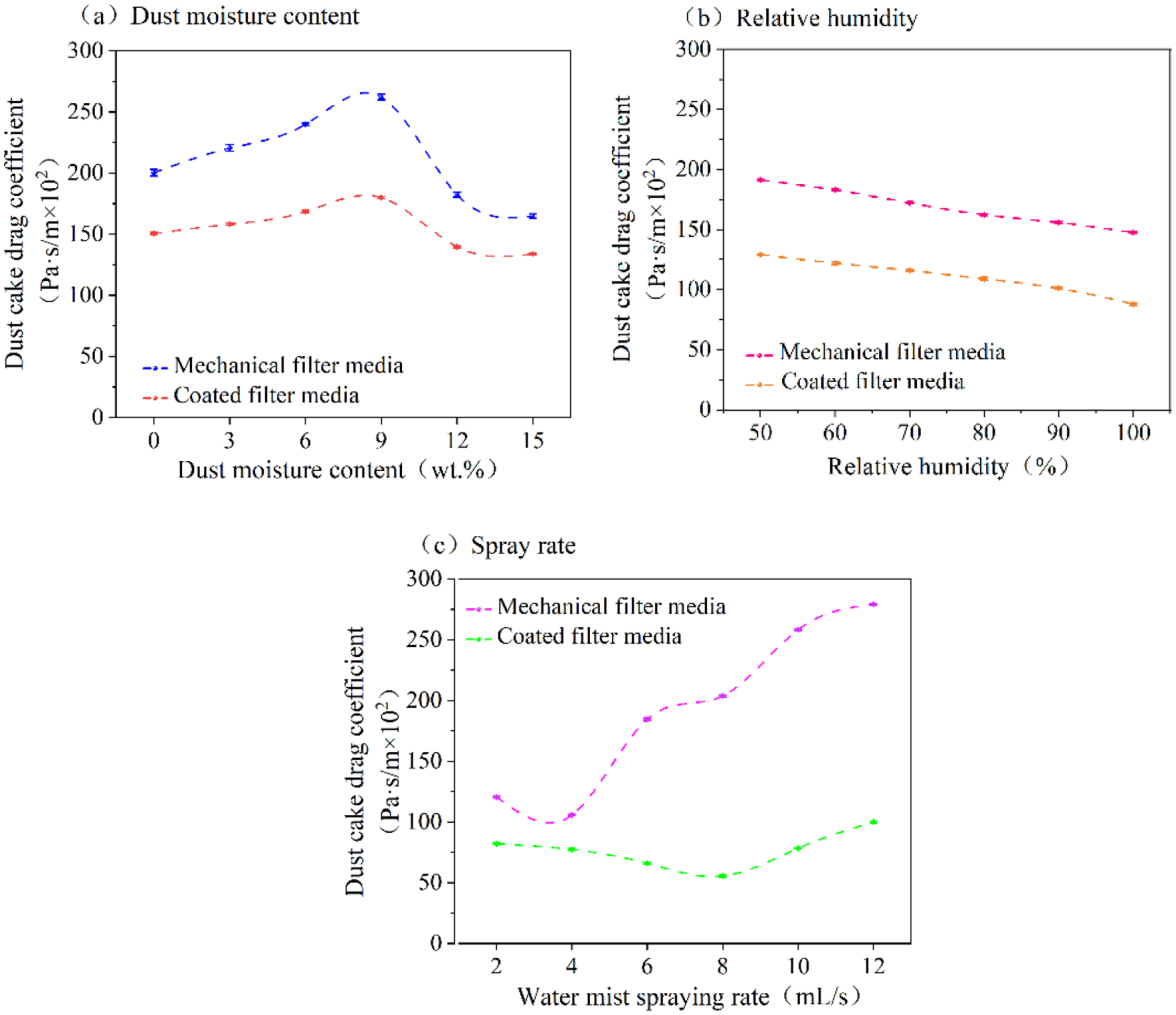
Dust cake drag coefficients variation of two filter media under different wet environments.

The same initial filtration velocity was set in this experiment. Dust cake drag coefficients was calculated using the average filtration velocity throughout the whole filtration process. Dust cake drag coefficients represents the pressure drop of the dust cake per unit filtration velocity. The variation in dust cake drag coefficients for the two filter media under different wet environments are presented in Fig. 5. The dust cake drag coefficients value of the mechanical filter media is higher than that for the coated filter media under the same wet environment. The change in dust cake drag coefficients for both filter media with varying dust moisture content is presented in Fig. 5a. When the dust moisture content increases, the dust cake drag coefficients initially rises and then decreases. When the dust moisture content is 8 wt%, the dust cake drag coefficients is the highest value for both filter media. The dust cake drag coefficients is lowest when the dust moisture content is 15 wt%. This behavior can be attributed to the increase in dust moisture content increases deposition, friction, and adhesion forces. When dust moisture content is lower, the change in friction and adhesion forces on the surface of dust particles is relatively small. Additionally, the change in deposition forces is greater than the changes in friction and adhesion forces, resulting in the formation of a compact dust cake with a higher compression coefficient. A higher dust moisture content (in the range of 8–15 wt%) can lead to changes in friction and adhesion forces on the surface of the particles become more significant compared to their deposition forces. As a result, the compression of the formed dust cake is reduced, leading to a looser structure. Consequently, the dust cake drag coefficients value is decreases.

The variation of the dust cake drag coefficients for mechanical filter media and coated filter media with relative humidity is presented in Fig. 5b. The dust cake drag coefficients of both filter media increases as relative humidity increases, reaching the maximum at an relative humidity of 50%. When the relative humidity is 100%, the dust cake drag coefficients value is minimal for both filter media. The increase in relative humidity enhances the probability of capillary coalescence between dust particles, leading to an increased formation of liquid bridges and greater adhesion of the dust particles to their surfaces. Consequently, the dust cake becomes looser, and the dust cake drag coefficients decreases.

The variation in dust cake drag coefficients for mechanical filter media and coated filter media with the spray rate is shown in Fig. 5c. As spray rate increases, the dust cake drag coefficients value for both filter media initially decreases and then increases. The dust cake drag coefficients for mechanical filter media is minimized at spray rate of 4 mL/s and maximized at 12 mL/s, and it rises rapidly in the range of 4–6 mL/s and 8–10 mL/s. For coated filter media, the dust cake drag coefficients is minimized at an spray rate of 8 mL/s and maximized at 12 mL/s. The spray rate corresponding to the lowest point of the dust cake drag coefficients for the coated filter media lags behind that of mechanical filter media. In addition, the maximum dust cake drag coefficients value for coated filter media is significantly smaller than that for mechanical filter media. As a result of the smooth surface of coated filter media, dust is less likely to adhere to its surface, resulting in a lower $\Delta {P}_{\text{C}}$ and a lower dust cake drag coefficients.

### The influence of wet environments on the dust loading capacity

**Figure 6:**
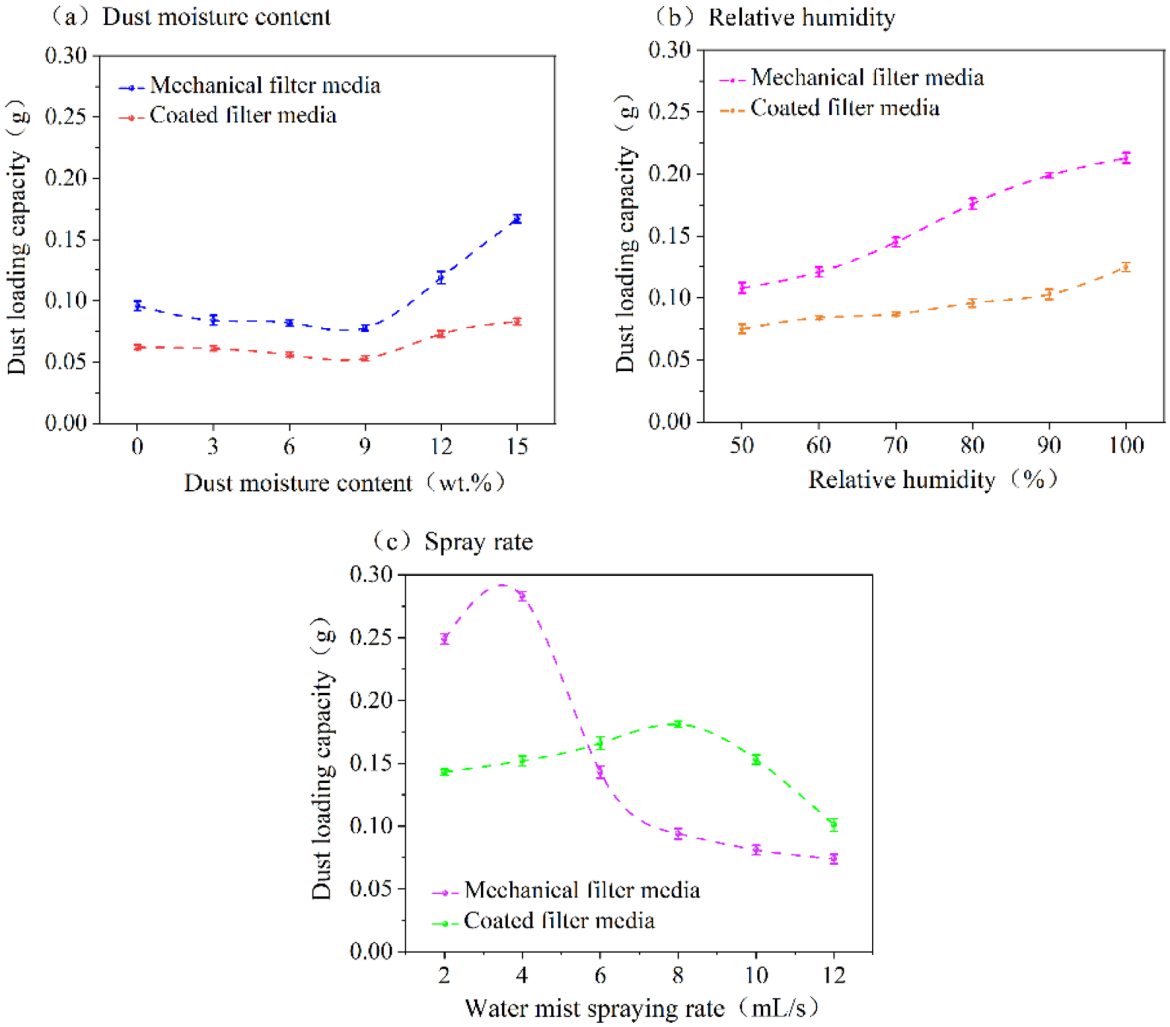
Dust loading capacity of two filter media under different wet environments.

The variation in dust loading capacity for both filter media under different wet environments is presented in Fig. 6. In Fig. 6a, the dust loading capacity of both filter media initially decreases and then increases with the increasing dust moisture content. For both filter media, dust loading capacity is minimized when the dust moisture content value is 9 wt% and maximized when the dust moisture content value is 15 wt%. In Fig. 6b, the dust loading capacity of both filter media increases with increasing relative humidity. At 50% relative humidity, the dust loading capacity of both filter media is maximized, and its maximum at 100% relative humidity. As shown in Fig. 6, the mechanical filter media exhibits a higher dust loading capacity compared to coated filter media under the effects of both dust moisture content and relative humidity. In both cases, the coated filter media performs better than mechanical filter media due to the microporous film coating on its surface.

As shown in Fig. 6c, dust loading capacity increases initially and then decreases for both filter media as the spray rate increases. The dust loading capacity of mechanical filter media is highest at 4 mL/s and lowest at 12 mL/s. For coated filter media, the dust loading capacity is greatest at 8 mL/s and least at 12 mL/s. The results can be attributed to the spray rate is low, the filter surface can form a looser dust cake structure, resulting in a lower pressure drop, reduced filtration velocity, and increased dust deposition. As the spray rate increases, liquid bridges between dust particles fill up a significant portion of the pore spaces within the dust cake. This leads to an increase in pressure drop within the filter media and a faster reduction in filtration velocity, thus reducing dust loading capacity. Furthermore, when the spray rate is in the range of 2–4 mL/s, the dust loading capacity of mechanical filter media is larger. This phenomenon can be attributed to the fact that the difference in filtration velocities between the two filter media is not significant. However, due to the rougher surface of mechanical filter media, dust particles adhere more strongly to its surface, resulting in a higher dust attachment. When the spray rate is in the range of 6–12 mL/s, the dust loading capacity of coated filter media is higher. This is because the higher spray rate causes the filtration velocity of coated filter media is greater than that of the mechanical filter media, and the difference is significant, causing more dust to adhere to the surface of coated filter media. A higher dust loading capacity for the filter media will result in more dust adhering to its surface under the same conditions of inlet dust concentration.

## Conclusion

An experimental system was constructed to investigate dust cake parameters. The effect of wet environments on collapse angles of dust particles, collapse angles between dust particles and filter media, dust cake drag coefficients, and dust loading capacity of filter media was examined. The conclusions have been drawn as follows.

1. The collapse angles of dust particles increases with the increase of dust moisture content, relative humidity, and spray rate. The collapse angles of dust particles and its increase relatively smaller in the dust moisture content condition, while they are greater in the spray rate condition.
2. In wet environments, the collapse angles between dust particles and filter media varies similarly to collapse angles of dust particles. Furthermore, the CADF-M is greater than CADF-C in various wet environments. The rate of change of CADF-C is relatively small as the wet environment changes.
3. As the dust moisture content increases, the dust cake drag coefficients of the two filter media initially increases and then decreases. The dust cake drag coefficients of the two filter media decreases with increasing relative humidity. With the increase in spray rate, the dust cake drag coefficients decreases and then increases. The dust cake drag coefficients and its variation are smaller when the wet environment is consistent, and this pattern is more prominent when spraying is involved.
4. The dust loading capacity of two filter media decreases and then increases with rising dust moisture content. It also increases with the increasing relative humidity, but it increases with spray rate and then decreases. Under specific conditions of dust moisture content and relative humidity, the dust loading capacity of mechanical filter media is higher than that of coated filter media, but the difference is relatively small. When spray rate is lower than 4 mL/s, the dust loading capacity of the mechanical filter media is superior to coated filter media. When spray rate exceeds 4 mL/s, the dust loading capacity of mechanical filter media becomes lower than that of coated filter media. The mechanical filter media with a higher dust loading capacity has a greater tendency to attract dust more readily than that of the coated filter media under the same dust concentration conditions.

### Supplementary Information

Supplementary Information.
