# Supplementary material for: Effects of various wet environments on the characteristics of the dust cake deposited on the surface of filter media
Source: Sci Rep. 2023 Oct 10;13:17120. doi: 10.1038/s41598-023-44429-4 (PMC10564904; doi:10.1038/s41598-023-44429-4)
Supplement: Supplementary file 1 — Supplementary Information. [file 41598_2023_44429_MOESM1_ESM.docx]

***Supporting Information***

**Effects of various humid environments on the** **characteristics of the dust cake deposited on the surface of filter media**

Shihang Li^1,2,3,*^ , Yihan Lin^1,2^, Muze Han^1,2^, Hao Liu^1,3^, Jiang Shao^3^, Xiaoyu Tan^1,3^, Yuchen Luo^1,3^ & Rongting Huang^3^

*^1^ Jiangsu Key Laboratory of Coal-based Greenhouse Gas Control and Utilization, Carbon Neutrality Institute, China University of Mining and Technology, Xuzhou 221008, China;*

*^2^ School of Environment Science and Spatial Informatics, China University of Mining and Technology, Xuzhou 221116, China;*

*^3^ Jiangsu Engineering Research Center of Dust Control and Occupational Protection, School of Safety Engineering, China University of Mining and Technology, Xuzhou 221116, China.*

**S1 Filter media**

Mechanical filter media and polytetrafluoroethylene (PTFE) coated filter media, were chosen for the experiment. The scanning electron microscope (SEM) images of both types of filter media are shown in Figure S1, and their corresponding performance parameters are listed in Table S1.

**
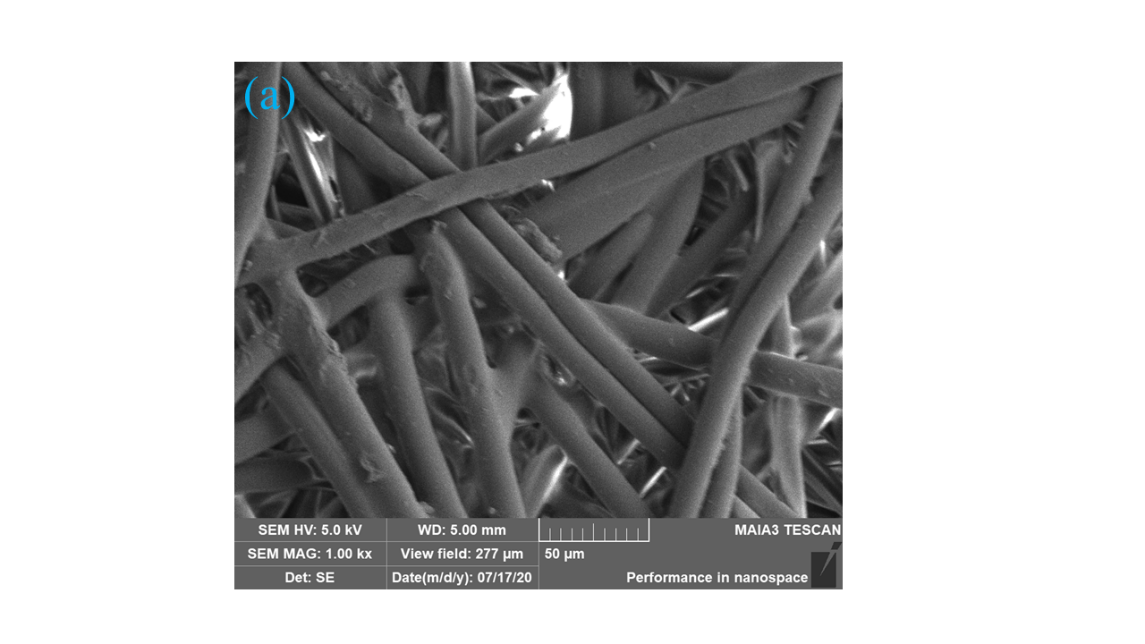

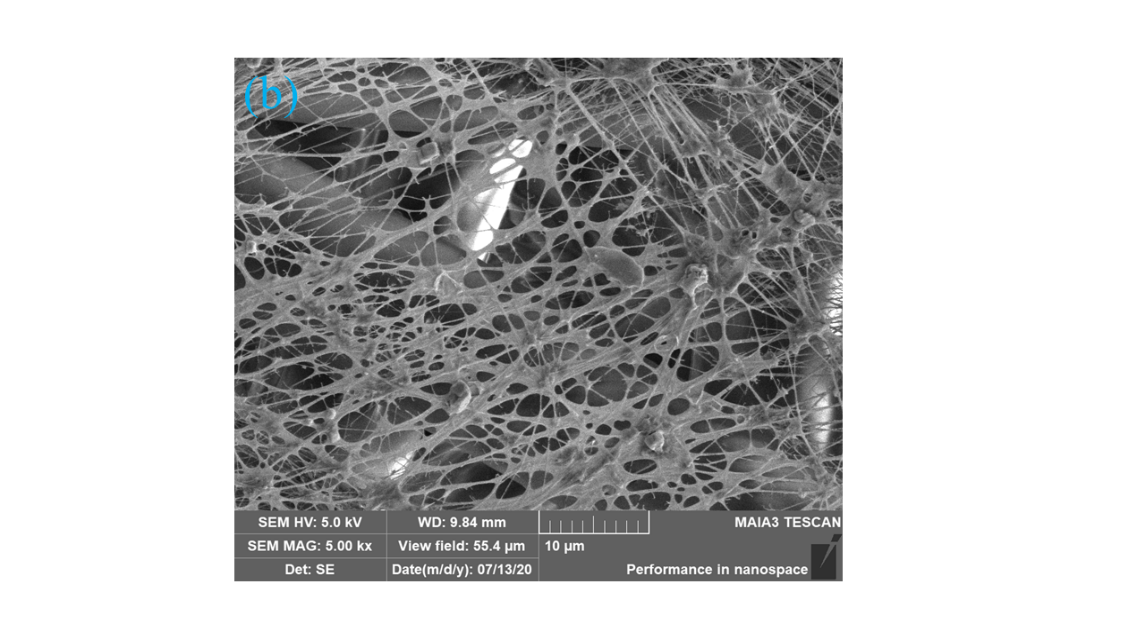
**

Fig. S1 SEM images of the surfaces of (a) mechanical filter media and (b) coated filter media

Table S1 Properties of the two types of filter media

| Parameters | Mechanical filter media | Coated filter media |
| --- | --- | --- |
| Surface treatment^*^ | Themo-bonding | Microporous coating |
| Surface mass^*^ (g/m^2^) | 240 | 255 |
| Thickness^*^ (mm) | 0.5 | 0.6 |
| Fiber diameter^*^ (um) | 15 | 0.2 |

*Provided by manufacturers

**S2 Setting up wet environments**

**S2.1 Dust moisture content**

After weighing six equal parts of fly ash (0% moisture content), a certain amount of water was added to five of them to obtain fly ash with dust moisture contents of 3 wt.%, 6 wt.%, 9 wt.%, 12 wt.%, and 15 wt.%. By weighing the quality of fly ash before and after adding water, we ensured that the error between the true value and the pre-set dust moisture content was less than 1%. Particle size distribution under different moisture contents was shown in Table S2. The water-containing dust was put into a aerosol generator. Subsequently, dust with a specific moisture content could be gained by turning on the aerosol generator and regulating Valve 3 (RV3).

Table S2 Particle size distribution under different moisture contents

| Moisture contents (wt.%) | D10 (μm) | D50 (μm) | D97 (μm) |
| --- | --- | --- | --- |
| 0 | 4.4 | 10.4 | 48.3 |
| 3 | 3.9 | 9.8 | 47.9 |
| 6 | 4.3 | 10.1 | 46.1 |
| 9 | 3.7 | 10.6 | 49.1 |
| 12 | 4.5 | 9.8 | 48.3 |
| 15 | 4.3 | 10.2 | 47.3 |

**S2.2 Relative humidity**

The relative humidity condition was regulated by a bubble humidifier. The relative humidity of the experimental system was measured by a hygrometer after 10 min of stable operation. Considering the high relative humidity in the underground space, the relative humidity was set to 50%, 60%, 70%, 80%, 90%, and 100%, respectively.

**S2.3 Spray rate**

An atomizer was used to produce spray in this experiment. The spray rate of 2 mL/s, 4 mL/s, 6 mL/s, 8 mL/s, 10 mL/s, and 12 mL/s were achieved by adjusting the atomizer.
